# Grease-gun injury of the orbit: two cases report and literature review

**DOI:** 10.1186/s12886-023-03032-x

**Published:** 2023-07-14

**Authors:** Wei Shen, Yang Yang, Yunshan Su, Zhulin Hu

**Affiliations:** 1 Department of Ophthalmology, Affiliated Hospital of Yunnan University, The Second People’s Hospital of Yunnan Province, Eye Hospital of Yunnan Province, 176 Qingnian Road, Kunming, China; 2 Department of Radiology, Affiliated Hospital of Yunnan University, The Second People’s Hospital of Yunnan Province, Eye Hospital of Yunnan Province, Kunming, Kunming China

**Keywords:** Trauma, Orbit, Foreign body, Grease-gun

## Abstract

**Background:**

The grease-guns injury is an uncommon injury to the orbit. We present the twelfth and thirteenth cases of grease-gun injury to the orbit to be reported in the English language literature since 1964. Here we discus and review the presentation, investigation, and treatment of this unusual trauma.

**Case presentation:**

Case 1 was a 29-year-old man who presented 1 day after a grease-gun injury of the left orbit with severe pain, marked periorbital swelling, and proptosis. Computed tomography (CT) revealed penetration of grease into his left orbit. Following surgical removal, proptosis decreased. The limitation of extraocular movement and loss of visual acuity to finger count was discovered after the initial surgery. Motility gradually returned. Visual acuity recovered after phacoemulsification, capsular tension ring and intraocular lens implantation for traumatic cataract and subluxation. Case 2 was a 6-year-old boy who was referred 2 months after a grease-gun injury for worsening swelling with sinus, necrosis and slight ptosis of the upper left eyelids. This is a case of orbital chronic inflammation from grease-gun injuries masquerading as orbital cellulitis. The imaging findings of CT and magnetic resonance imaging (MRI) are not typical. Surgical exploration and debridement was inevitable and actually relieved the symptoms.

**Conclusions:**

Grease-gun injuries can damage the orbit in different degrees. Careful history inquiry and taking is important to establish the diagnosis. Imaging examinations using CT or MRI are helpful to determine depth of trauma and foreign bodies in the orbit at diagnosis. We suggest that surgical exploration and debridement is a key step in the management.

## Background

Grease-gun injuries have been reported in various parts of the body including hands, chest, genitals, and, in rare instances, in the eye and its adnexa [1]. To our knowledge, only 11 cases involving the orbit have been published since 1964 [2–11]. We described two cases who suffered grease-guns injury to the orbit.

## Case presentation

### Case 1

A 29-year-old man sought treatment 1 day after accidental injection of grease in his left orbit from a high-pressure hydraulic machine while repairing his excavator. On presentation, he complained of increasing pain and marked swelling and proptosis of the left eye.

**Fig. 1:**
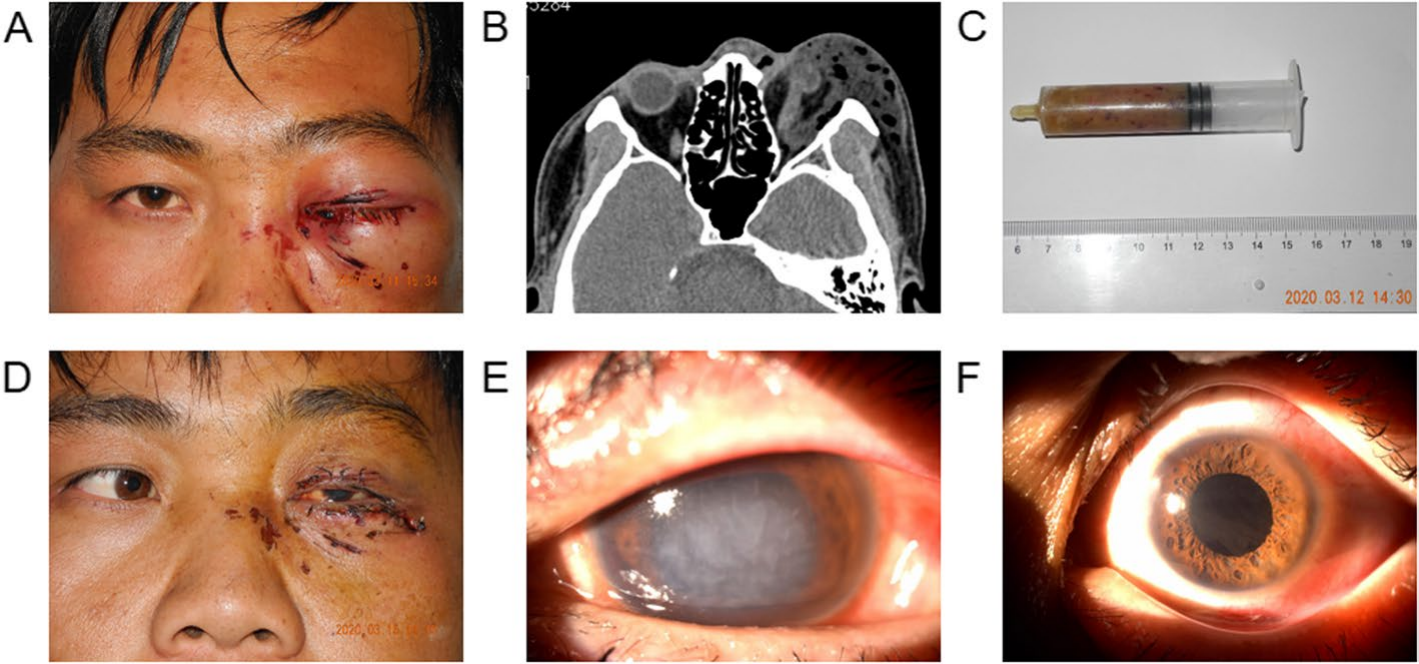
Case 1. **A** Appearance on presentation showing marked periorbital swelling with irregular wound, blood scab and proptosis. **B** Orbital CT showing significant axial proptosis and extensive inflammation with numerous bubble-liking hypodense masses within the left orbital and periorbital soft tissue. **C** The grease removed from the orbit. **D** Appearance on the third day after the initial surgery showing reduced periorbital swelling and proptosis with a remarkable restriction of abduction of the left eye. **E** The view of the anterior segment of the left eye on the 3rd day after the initial surgery showing corneal edema, dilated pupil, lens opacity. **F** The view of the anterior segment of the left eye on the 7th day after the second surgery

Examination: The right eye was found to be normal and the visual acuity 0.6 unaided. The left eye cannot complete the vision test. There was marked periorbital swelling with irregular wound, blood scab and proptosis (Fig. 1A), conjunctiva with hyperemia and edema, cornea with edema and turbidity, and unclear intraocular structure. General physical examination, body temperature, and chest radiography were also within normal limits. The white blood cell count was14.99 × 10^9^/L. The neutrophil count is 13.10 × 10^9^/L.

Orbital Computed tomography (CT) showed significant axial proptosis and extensive inflammation with numerous bubble-liking hypodense masses within the left orbital and periorbital soft tissue (Fig. 1B).

The patient was diagnosed with orbital foreign bodies, eyelid injury and eyeball contusion.

Systematic high-dose corticosteroid treatment (intravenous injection methylprednisolone, 1 g, three days), systematic antibiotics (intravenous injection cefuroxime sodium, 0.75 g, three days), and topical antibiotics and corticosteroid (Tobramycin and Dexamethasone Eye Drops, q.i.d.) began in the first hospital day. Surgical exploration and debridement in his left orbit was performed under general anesthesia through an eyelid crease approach on the second hospital day. A large amount of thick yellow greasy foreign bodies were found throughout the subcutaneous tissue and orbital fat (Fig. 1C), and these were subsequently removed as much as possible. The surgery went smoothly. A drain was inserted in the inferior orbit for 72 h.

Three days after surgery, his left periorbital swelling was significantly improved (Fig. 1D). However, his visual acuity was counting fingers at 1 m in the left eye. Extraocular movements (EOM) revealed a remarkable restriction of abduction of the left eye. There was corneal edema, dilated pupil, lens opacity, and unclear fundus (Fig. 1E).

After another five months, the patient complained of decreased visual acuity in the left eye. Visual acuity was hand motions in the left eye. EOM was improved. Slit lamp examination showed lens opacity and subluxation. He underwent phacoemulsification, capsular tension ring and intraocular lens implantation.

One week after the second operation, his uncorrected visual acuity was 0.5 in the left eye (Fig. 1F). Intraocular pressure was 16 mmHg in the left eye. Fundus examination was normal. Follow-up was unremarkable, and the patient’s condition is stable.

### Case 2

A 6-year-old boy sustained an injury to the left eye with a jet of oil from a grease-gun. He was not referred to our hospital until 2 months after the accident with worsening left periorbital swelling. The next day after the injury, the child developed swelling and pus on the upper eyelid of the left eye, difficulty in opening his eyes, and obscured vision. After the injury, the patient went to two local hospitals, treated with broad spectrum intravenous antibiotics. One month after the injury, the swelling of the left eyelid gradually increased and the area around the wound became black.

**Fig. 2:**
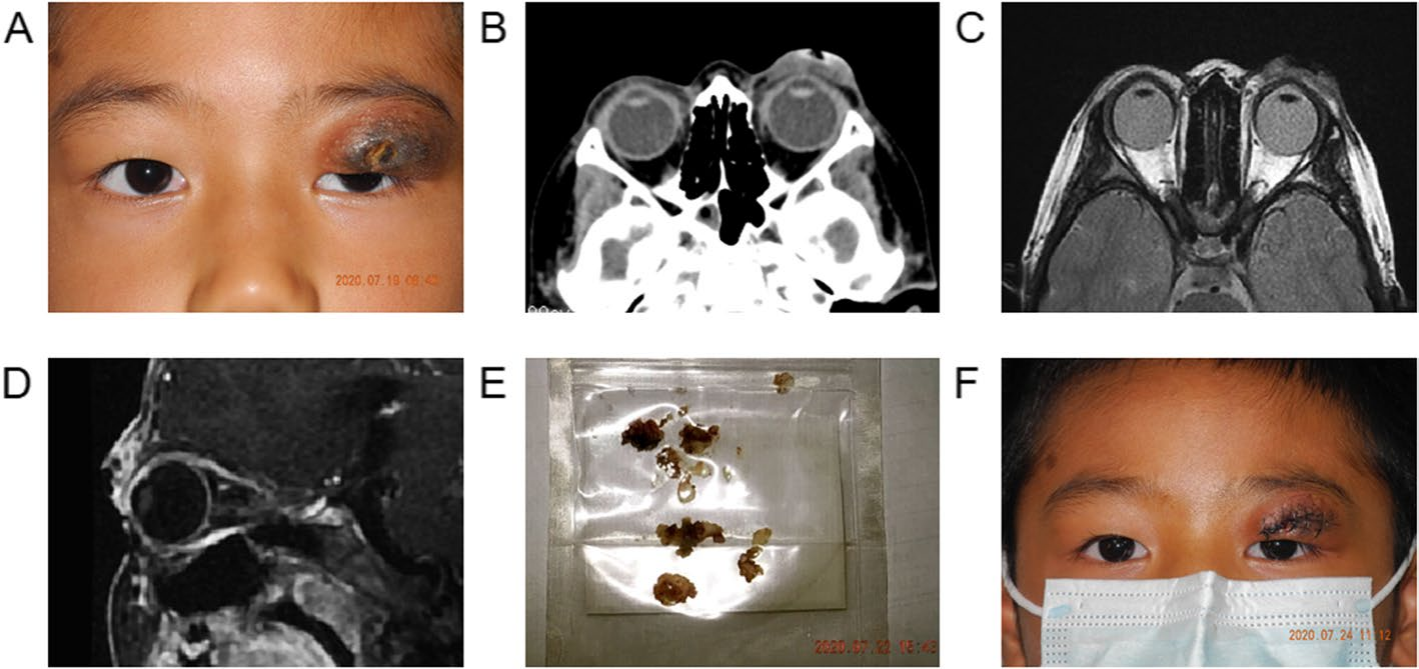
Case 2. **A** Appearance on presentation showing swelling of the left upper eyelid with a chronic discharging sinus, necrosis and slight ptosis. **B** CT scan of the orbits showing extensive swelling and increased density of the subcutaneous tissue of the left upper eyelid. **C** On T2 axial and sagittal plain MRI, the left upper eyelid was irregularly thickened, showing mixed and slightly hyperintensity, and the edge was unclear. Small strips of hypointensity were seen in the anterior part of the lesion, and the edge was unclear. **D** On sagittal enhancement of T1, uneven and obvious enhancement was seen in the left upper eyelid area, and no enhancement was seen in the anterior hypointensity area of the lesion, and the edge was clear. **E** Solidified grease and abnormal tissue surgically removed from the the orbicularis and orbital fat through the palpebral incision. **F** Appearance on the seventh day after surgery showing reduced swelling and ptosis of the left upper eyelid

Examination: The right eye was found to be normal and the visual acuity 0.6 unaided. In the left eye the visual acuity was 0.4 unaided. There was a swelling of the upper eyelid with a chronic discharging sinus, necrosis and slight ptosis (Fig. 2A). General physical examination, body temperature, and chest radiography were also within normal limits. The white blood cell count was 4.35 × 10^9^/L. The neutrophil count is 1.75 × 10^9^/L. The lymphocyte count is 2.17 × 10^9^/L.

CT scan of the orbits showed extensive swelling and increased density of the subcutaneous tissue of the left upper eyelid (Fig. 2B).

On axial and sagittal T2 weighted magnetic resonance imaging (MRI) (Fig. 2C), the upper eyelid was irregularly thickened, showing mixed and slightly hyperintensity, and the edge was unclear. Small strips of hypointensity were seen in the anterior part of the lesion, and the edge was unclear. On enhanced sagittal T1 weighted MRI (Fig. 2D), uneven and obvious enhancement was seen in the upper eyelid area, and no enhancement was seen in the anterior hypointensity area of the lesion, and the edge was clear.

The boy was initially diagnosed with orbital cellulitis, but the orbital foreign body could not be excluded.

An orbital exploration of the left eye was arranged. The boy underwent surgical exploration under general anesthesia via a left palpebral incision around the sinus and necrosis. Intraoperationally, solidified yellow greasy foreign bodies were found throughout the orbicularis and orbital fat and this was subsequently removed (Fig. 2E); the abnormal tissue was debrided. The surgery is very difficult.

After orbital exploration and debridement, he received 1 g of intravenous injection methylprednisolone and 0.75 g of intravenous injection cefuroxime sodium for 3 days followed by a 2-week oral prednisone taper.

The boy responded rapidly to treatment with preserved visual function and substantially improved periorbital swelling, motility, and proptosis (Fig. 2F). The patient has been lost in follow-up.

## Discussion and conclusions

Our literature search was performed in the PubMed database using the text words (“grease-guns” OR “hydraulic oil”) AND (“orbit” OR “eyelid” OR “face”). In the review of the accessible English literature, only 11 cases of grease-gun injuries to the orbit have been reported [2–11].

Grease guns are common workshop tools used to apply grease to rotating parts in heavy-duty vehicles to be lubricated under high pressure. The special nature of grease-gun injury depends upon two main factors-physical distension and chemical irritation [1]. Grease, which has a high viscosity and low toxicity and contains a calcium, sodium or lithium-soap jelly emulsified with mineral oil, is the least destructive material. However, greases with the high pressure can result in focal penetration followed by quickly diffusing along fasciae, tendons, skeletal muscles, and neurovascular bundles to a considerable distance [1]. The chemical irritation of the substance may cause the tissue reaction which is of a slow granulomatous inflammation in the dermis and subcutaneous tissue, but may lead to massive fibrosis, encysted collections of oil, tissue necrosis, and sinus formation at a late stage and account for much loss of function [1].

Grease-gun injuries of the orbit occur very rarely. The clinical characteristics of the previous and present case series are summarized in Table 1. These cases demonstrate the rare injuries generally occur in males. The observed age ranges from 6 to 65 years. The main clinical manifestations were swelling of the periorbital or eyelids (12/13), proptosis (8/13), pain (8/13), decreased vision (7/13), limited EOM and diplopia (7/13). Visual acuity at presentation ranges from 20/20 to no light perception. Injuries usually involve the left eye except two cases. It is conventionally speculated that the grease gun is usually held in the right hand, which is the dominant hand for most people. Patients were usually treated in emergency departments immediately following injury, and then referred to other specialist medical institution for further treatment. Some patients had to visit many hospitals because of no improvement.

**Table 1:** Clinical profiles of 13 cases with grease-gun injury of the orbit

Author	Year	Sex	Age	Injured eye	Country	Time-to-operation	Clinical manifestation	Preoperative VA	CT	MRI	Management	Postoperative VA
Dallas NL [2]	1964	Male	39	left	UK	3 months	bruising and swelling of the lids and conjunctiva, ptosis, proptosis, limited EOM, congestion of the retinal veins, swelling of the optic disc, and visual field defect	6/12	/	/	Long term steroid treatment; orbital exploration and debridement	6/12
Boukes RJ, et al. [3]	1987	Male	19	left	Netherlands	3 days	swelling of the lids, reduced VA, proptosis, pain, visual field defect	LP	+	+	Drainage, irrigation, and aspiration	1.0
Woher JR, et al. [4]	1991	Male	32	left	USA	2 days	edema of eyelids, eyebrow, and forehead	20/20	+	/	Exploration, irrigation, and drainage; Scar revision	20/20
Goel N, et al. [5]	1994	Male	18	right	Canada	not available	loss of VA, ptosis, and ruptured globe	NLP	+	/	Enucleation and debridement; Intranasal ethmoidectomy, sphenoidectomy, inferior turbinectomy and antrostomy	NLP
Goel N, et al. [5]	1994	Male	25	left	Canada	About 4 weeks	Pain, periorbital bruising and swelling, proptosis, diplopia, limited EOM	20/40	+	/	Anterior orbitotomy	20/20
Gekeler, et al. [6]	2005	Male	31	left	Germany	11 months	pain, reduced VA, edema of eyelids, and vitreous hemorrhage	20/40	+	+	Conservative care; Surgical removal (at the patient’s request)	20/20
Bar T, et al. [7]	2005	Male	39	left	Israel	some hours	swelling of the left side of the face, periorbital haematoma, and pseudoptosis	/	+	/	Irrigation and debridment	/
Wang Y, et al. [8]	2008	Male	44	right	China	20 days	pain, orbital swelling, and decreased VA, proptosis, ptosis, limited EOM, diplopia, posterior choroidal folds, retinal vein dilation, and papilledema with blurry margins	20/200	+	+	Orbital exploration	20/40
Park JH, et al. [9]	2010	Male	48	left	Korea	1 day	decreased VA, pain, swelling and abrasions of the eyelids, limited EOM, proptosis, lens subluxation, and high IOP	HM	+	/	Orbital exploration and debridement; Vitrectomy, lensectomy and intraocular lens implantation	0.1
Cheema M, et al. [10]	2018	Male	65	left	Canada	8 days	periorbital swelling, diplopia, limited ductions, and proptosis	20/60	+	/	Orbital biopsy and debridement	20/60
Chakraborti C, et al. [11]	2020	Male	20	left	India	5 days	dimness of vision, pain, swelling of lids, proptosis, limited EOM, corneal abrasion, and traumatic optic neuropathy	CF	+	/	Anterior orbitotomy	20/20
Our case 1	Present study	Male	29	left	China	1 day	Pain, periorbital swelling, proptosis, dcreased VA, limited EOM, corneal edema, dilated pupil, lens opacity and subluxation	CF	+	/	Orbital exploration and debridement; Phacoemulsification, capsular tension ring and intraocular lens implantation	1.0
Our case 2	Present study	Male	6	left	China	2 months	Swelling of the lid with sinus, necrosis and slight ptosis	0.4	+	+	Long term antibiotics treatment; Orbital exploration and debridement	0.4

*VA* vision acuity, *LP* light perception, *NLP* no light perception, *HM* hand motions, *CF* counting finger, *EOM* extraocular movement, *IOP* intraocular pressure

As the type of foreign body is unfamiliar, grease-gun injuries of the orbit might deliver some diagnostic and therapeutic problems.

An immediate diagnosis was somewhat difficult. Since initial presentation may be deceptive, treatment is frequently delayed. Some grease-gun injuries may cause penetrating foreign body injuries without an obvious or visible entry wound in the skin, especially when the initial small lesion has healed over in later presentations [1, 5]. This was merely the tip of the iceberg since subcutaneous tissue was damaged far more severely than this. If external wounds or ocular symptoms are not severe, rare foreign bodies may be underestimated. If the inflammation around the orbit is severe, the injuries may be initial diagnosed as orbital cellulitis, such as the case reported by Dallas NL [2], Boukes RJ et al. [3], Cheema M et al. [10] and our case 2. Careful history inquiry and taking is important to establish the diagnosis. The application of CT and MRI enabled accurate delineation of deep trauma, localization and tissue recognition of foreign bodies in the orbit [3, 5]. An orbital CT shows typically as numerous or isolated bubble-liking hypodense masses within the orbit, often accompanied by extensive inflammation and proptosis [3–11]. MRI shows the presence of soft tissue masses with high signal intensity area centrally and the wall of lower signal intensity resembling tissue reaction within the orbit. On T1 weighted MRI significant differences between retrobulbar fat and grease were found. On T2 weighted MRI, the differences between signal intensities were hardly apparent [3]. However, the imaging findings of CT and MRI in a few cases are not typical. In our case 2, enhanced T1 showed uneven and obvious enhancement in the upper eyelid area resembles grease, and no enhancement in the anterior hypointensity area of the lesion resembling tissue reaction.

There are different opinions on the choice of treatment for grease-gun injuries of the orbit. Most authors submitted to surgical exploration and debridement immediately [1–5, 7–11]. Surgical exploration can confirm the rare foreign bodies in the orbits. However, Gekeler et al. reported a case of an intraconal grease cyst that was followed for 11 months without symptoms, and suggested that small amounts of intraconal deposition of oily substances can be carefully observed for extended periods of time and may not necessarily require surgical intervention [6]. The consequence of incomplete debridement is the formation of chronic lipogranulomas, tissue necrosis or sinus tracts, such as the case reported by Gekeler et al. [6], the case reported by Wolter JR and Nelson CC [4], and our case 2. In fact, patients often underwent one or more surgical treatments after injury. Most patients had good visual outcome after operation, except for cases with eyeball injury or optic nerve injury.

Our cases emphasize the spectrum of grease-gun injuries to the orbit. Case 1 was a typical case with clinical manifestations and imaging features. Surgical exploration and debridement was timely and effective. In addition to trauma to the orbit, the patient suffered an eyeball contusion. Visual acuity recovered after a second surgery for traumatic cataract and subluxation. In the early post-injury period, the eyeball injury may not be assessed timely and accurately because of marked periorbital swelling. The eyeball damage should be followed-up persistently even if it is not presently troublesome. Case 2 was a case of orbital chronic inflammation from grease-gun injuries masquerading as orbital cellulite. The imaging findings of CT and MRI are not typical. Atypical features without satisfactory response to appropriate empiric antibiotics should prompt surgical exploration. Surgical removal actually relieved the symptoms. The case emphasizes that grease and necrotic tissue must be early debrided with surgical exploration, which may prevent healing.

In conclusion, the previous and present cases demonstrate grease-gun injuries can damage the orbit in different degrees. Careful history inquiry and taking is important to establish the diagnosis. Imaging examinations using CT or MRI are helpful to determine depth of trauma and foreign bodies in the orbit at diagnosis. We suggest that surgical exploration and debridement is a key step in the management of cases presenting similarly to ours. High-pressure grease guns can cause damage to any parts of the body. Safety training must be provided for workers, who handle grease guns, and information must be given about the necessity of using protective glasses, gloves and clothing and the potential dangers associated with grease guns.

## Data Availability

The datasets generated during and/or analyzed during the current study are available from the corresponding author on reasonable request.

## Abbreviations

CT: Computed tomography
MRI: Magnetic resonance imaging
EOM: Extraocular movements
